# Residential Characteristics as Factors Related to Healthy Behavior Practices—Decision Tree Model Analysis Using a Community Health Survey from Korea

**DOI:** 10.3390/ijerph19127390

**Published:** 2022-06-16

**Authors:** Ae-Rim Seo, Ki-Soo Park

**Affiliations:** 1 Department of Preventive Medicine, Institute of Health Science, College of Medicine, Gyeongsang National University, Jinju 52727, Korea; sarim2101@naver.com; 2 Center for Farmer’s Safety and Health, Gyeongsang National University Hospital, Jinju 52727, Korea

**Keywords:** healthy behaviors, residential characteristics, decision tree

## Abstract

In this study, we sought to identify relevant factors in healthy behavior practices, including not only individual-level variables but also regional and physical environments. Data from the Korea Community Health Survey (KCHS) of Gyeongsangnam-do in 2018 were used, with data from 16,519 of the 17,947 individuals (excluding 1428 individuals who had missing values) who participated in the survey. Healthy behavior practices were defined as meeting the criteria for all three modifiable healthy behaviors (non-smoking, moderate alcohol consumption, regular walking). A decision tree analysis was performed. In men, healthy behavior practices were lower in the unemployed population, in those aged 40–50 years, living in rural residential areas, and with stress. For women who lived in areas with small populations (<100,000 population), healthy behavior practices were below-average. Men and women who had below-average healthy behavior practices reported poor access to places for exercise and fair or poor self-rated health statuses. It is necessary to implement a health behavior practice intervention that considers not only individual characteristics but also access to local exercise facilities and residential area characteristics (urban, rural). Since age is an important variable in healthy behaviors for both men and women, customized programs that consider age should be provided.

## 1. Introduction

In recent years, the importance of the prevention and management of chronic diseases has increased. According to the World Health Organization (WHO), non-communicable diseases (NCDs), that is, cardiovascular disease, cancer, and diabetes caused 71% of global deaths in 2016 [1]. To achieve the target of the sustainable development goals (SDGs) of a one-third reduction in premature deaths from NCDs by 2030, it is necessary to modify the following risk factors for NCDs: tobacco use and high-risk drinking, lack of physical activity, and an unhealthy diet [1].

Smoking, high-risk drinking, and lack of physical activity are risk factors for poor health, but improving them can prevent or delay the worsening of chronic diseases [2]. Engaging in multiple risky behaviors is associated with a greater risk of chronic disease and mortality as compared with engaging in one or no risk behaviors [3,4,5]. In addition, mortality risk and the risk of poor health-related quality of life increase when the number of poor behaviors increases [6,7,8,9].

An increasing number of studies have explored the clustering of health behaviors [6,10,11]. Clustering at both ends of the spectrum has been reported, with larger numbers of individuals than expected exhibiting all or none of a range of risk factors [6,11,12,13].

Therefore, given that risk behaviors rarely occur in isolation, tackling multiple rather than single behaviors may be a more effective approach [3]. Multiple-behavior change interventions may therefore have a greater potential for causing a positive impact on health outcomes than single-behavior change interventions [6,14,15].

Health behaviors are influenced in a complex way by an individual’s demographic characteristics, health status, and the characteristics of the region in which they live.

Population-based health promotion through health interventions is needed to improve healthy lifestyles. However, so far, the primary focus has been on high-risk-based health promotion interventions that find and modify individual risk factors. To carry out health promotion interventions, it is important to select subjects with various risk factors. Recently, in the field of public health, the decision tree analysis has been widely used as a useful method of classifying subjects with risk factors [16].

Decision tree analysis is a method of classifying the entire group into several subgroups and making a prediction by expressing the decision rule in the form of a tree structure. Because the process of classification and prediction describes the model in a tree structure, interpretation is easy, and it provides meaningful knowledge. In other words, since an accurate analysis of the target group is possible and provides a basis for specific classification, prediction, and policy development for the target group [17,18], the selection of health promotion program targets (group profiling) is advantageous. Recently, this analysis has been applied in various ways, such as in metabolic syndrome prediction [19] and in the health management behavior of diabetic patients [20]. However, most studies so far have included individual-level variables, and studies that included regional (including environmental) characteristics have been rare.

The purpose of this study was to identify factors relevant to healthy behavior practices that include not only individual-level variables but also regional (including environmental) variables, and to find the basis for the goal of improving health behaviors and the selection of the priority population. Ultimately, it was intended to be used as evidence for the need to bridge the health gap.

## 2. Materials and Methods

### 2.1. Subjects

#### Data Collection and Subjects

Data from the Korea Community Health Survey (KCHS) for Gyeongnam province in 2018 were used. The KCHS study [21] is a repeated cross-sectional study that has been conducted annually by the Korea Center for Disease Control and Prevention (KCDC) on about 900 residents for each of the 252 districts since 2008. The purpose of this survey is to produce regional representative statistics in Korea; its target population is Korean adults in general, aged 19 years or above. All of the surveys are conducted by trained interviewers that visit the sample households, using a face-to-face computer-assisted personal interviewing (CAPI) method.

**Figure 1:**
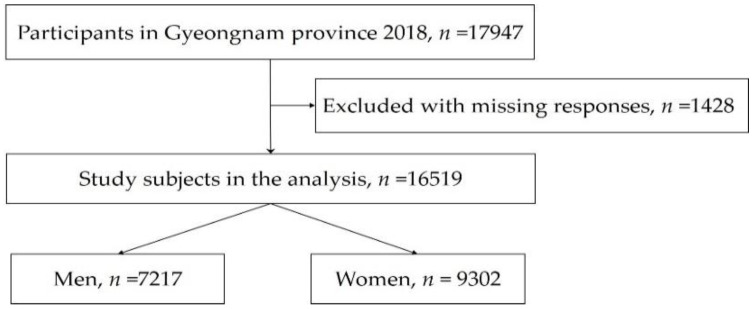
Flowchart of the study subjects.

The current study utilized 16,519 of the 17,947 individuals who participated in the KCHS in the Gyeongnam province in 2018, after excluding 1428 individuals who had missing values (individuals with missing data on the questionnaire were excluded) (Figure 1).

The current study was approved by the Gyeongsang National University Institutional Review Board (GIRB-A20-X-0063).

### 2.2. Materials

#### 2.2.1. Dependent Variables

All data were based on self-reported information. We examined three modifiable healthy behaviors in this study: non-smoking, not high-risk drinking, and regular walking. The subjects were divided into two groups according to whether or not they engaged in each healthy behavior.

Healthy behavior practices were defined as meeting the criteria for all three modifiable healthy behaviors (non-smoking, not high-risk drinking, and regular walking).

Non-smokers were defined as individuals who did not smoke at the time of the study (including former smokers); those who had never smoked or had smoked less than 100 cigarettes in their lifetime were considered non-smokers; and former smokers were defined as those who had smoked at least 100 cigarettes in their lifetime but were not smokers at the time of the survey. Current smokers were defined as those who had smoked five packs (100 cigarettes) or more in their lifetime and were currently smoking every day or on occasion.

Not-high-risk drinking was defined using the KCHS definition of high-risk drinking, which measures the amount of alcohol consumption through the number of drinks/day and the frequency of drinking; thus, since one standard drink in Korea contains 7–8 g of ethanol, high-risk drinking in Korea is defined as two or more drinking occasions per week, with more than seven (for men) or five (for women) standard drinks per drinking occasion. Those who did not meet this threshold were considered not-high-risk drinkers.

The definition of regular walking exercise was walking for at least 30 min per day and at least 5 days per week. Walking activity was measured through the following two questions: (1) “How many days did you walk for at least 10 min at a time in the last week? This includes at work and at home, walking to travel from place to place, and any other walking that you managed to do solely for recreation, sport, exercise, or leisure”, and (2) “How much time did you typically spend walking on each of those days?”

#### 2.2.2. Variables Related to Healthy Behavior Practice

In this study, demographics, health status, and regional variables were used as relevant factors for healthy behavior practices. The demographic variables collected were age, gender, monthly household income, education level, job, and marital status. Age was classified into 19–29, 30–39, 40–49, 50–59, 60–69, and ≥70 years. Monthly household income was classified as <2.0 and ≥2.0 million Korean won (KRW) (KRW 1.0 million is approximately USD 1000). Educational level was classified as middle school graduate or less and high school graduate or higher. Marital status was classified as living together (married/living with partner) or living alone (divorced/separated/widowed or never married).

Health status variables included body mass index (BMI), stress, experience of depression, self-rated health status (SRH), and the presence of hypertension and diabetes. BMI was defined using self-reported height and weight, and it was divided into three groups: BMI < 18.5 kg/m^2^ for underweight, BMI ≥ 18.5 and <25 kg/m^2^ for normal weight, and BMI ≥ 25 kg/m^2^ for obese. SRH was classified as good, fair, or poor. For the mental health variables, stress was classified as high for those who felt “very much” or “a lot” of stress in daily life, and low for those who did not. Perceived depression symptoms were defined as experiencing a feeling of depression that interfered with daily life for at least two consecutive weeks during the past year. High blood pressure and diabetes mellitus were marked with a “yes” if the subjects answered that they were suffering from these ailments, and “no” if they did not suffer from them.

Regional variables included satisfaction with house type, urban or rural area, city size according to the population size, and access to places for exercise; the atmospheric environment and green space were perceived as environmental factors. Housing types were divided into apartments and houses. The administrative district units in Korea have three structures: parish (*si/do*), county (*si/gun/gu*), and town (*eup/myeon/dong*). Each residential area was defined as urban or rural by classifying “*dong*” as an urban area, and “*eup/myeon*” as a rural area. In addition, city size was categorized based on the administrative districts with a population size of city 1 (≥300,000 population), city 2 (<300,000 and ≥100,000 population), city 3 (<100,000 and ≥50,000 population), and city 4 (<50,000 population). Access to places for exercise was defined as being able to easily find a place to exercise within one’s neighborhood in the past year. The perceived environment was divided into good and bad after participants evaluated their satisfaction with the atmosphere and the green space environments of the area in which they currently resided on a 5-point Likert scale.

### 2.3. Statistical Analysis

As for the general characteristics of the subjects, healthy behavior practice variables, including smoking and drinking, were divided by gender because there were differences according to gender. A chi-square test was performed and a *p*-value < 0.05 was considered to be statistically significant. Statistical analysis was performed using the SPSS version 25.0 (IBM Corp., Armonk, NY, USA).

**Figure 2:**
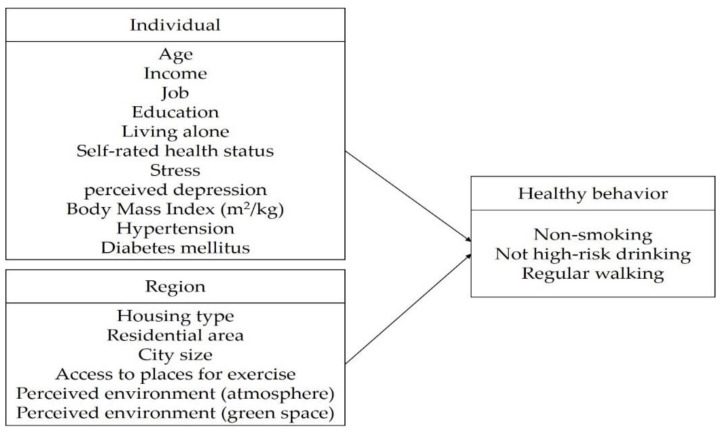
The analytic frame used to identify the relevant factors in healthy behavior practices.

A decision tree analysis was performed. This analysis was used to classify the group after using health behavior practices as dependent variables. Groups were classified using the decision tree model, and the feature importance of variables determining the health behavior practice groups were calculated, which helped in prioritizing variables to improve health behavior practices (Figure 2). Decision tree analysis was performed using Python (Python 3.7).

## 3. Results

### 3.1. General Characteristics of the Subjects

Of the total number of subjects, 9302 were women (56.3%). With regard to the demographic variables, those in their 70s or older accounted for the highest number of participants (27.0%), those who earned KRW 2 million or more accounted for 58.5%, while 60.4% were employed. In terms of the level of education, 68.2% graduated from high school or higher. In total, 67.6% lived together (married/living with partner). For the health status, 30.7% had good self-rated health, 20.4% had high stress, and 5.0% had perceived depression symptoms. According to the BMI, 34.9% of the participants were obese. In total, 29.3% of the patients were diagnosed with high blood pressure and 11.2% were diagnosed with diabetes mellitus. For the regional variables, 67.1% were living in houses and 65.1% resided in rural areas, 74.9% had easy to access to places for exercise, 66.6% had a good perceived atmosphere in their living environment, and 70.4% had a good perceived green space environment (Table 1).

There was a significant difference between the men and women in the demographic variables of age, income, job status (employed), education level, and their marital status (married/living with partner) (*p* < 0.001). SRH, stress, perceived depression symptoms, BMI, and hypertension were significant (*p* < 0.001). With regard to regional variables, housing type and access to places for exercise were significant (*p* = 0.011, *p* = 0.010). Diabetes mellitus, residential area (“*dong*” as urban area and “*eup/myeon*” as rural area), city size (the administrative districts with the population size), and perceived environment (atmosphere, green space) were not significant (Table 1).

The healthy behavior practice group included 29.2% of the total participants (Table 1). There was a significant difference in the percentage of healthy behavior practices according to sex (22.0% for men and 34.7% for women) (Table 1).

### 3.2. Decision Tree Analysis Results

#### 3.2.1. Results of the Decision Tree Analysis for Healthy Behavior Practices (Men)

**Figure 3:**
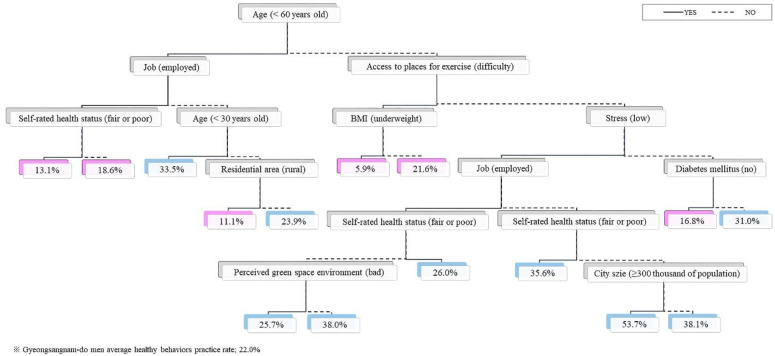
Decision tree analysis for healthy behavior practices (men).

The group of participants that was over 60 years old and had difficulty accessing places for exercise had the lowest level of healthy behavior practices (regardless of BMI: underweight (5.9%), normal weight and obesity (21.6%)), and 11.1% of the group had no job, were in their 40s up to 50s, and lived in rural areas (*eup/myeon*). In the group of participants who had a job, were under the age of 60, and had a fair SRH status, the practice of healthy behavior was 13.1%. In addition, healthy behavior practices were low in the group of participants who were over 60 years old, had easy access to places for exercise, high stress, and no diabetes (16.8%) (Figure 3).

#### 3.2.2. Results of the Decision Tree Analysis for Healthy Behavior Practices (Women)

**Figure 4:**
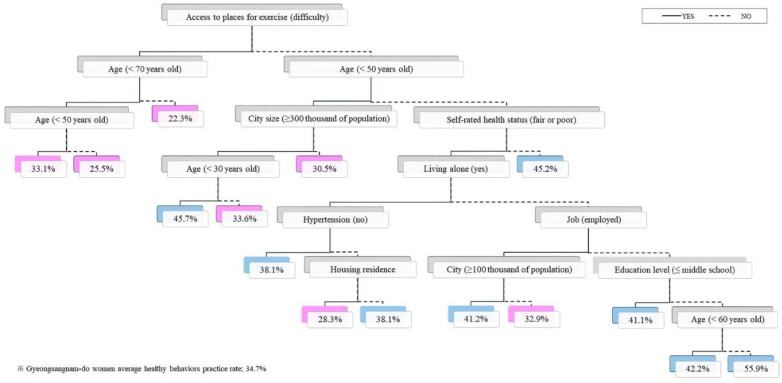
Decision tree analysis for healthy behavior practices (women).

When it was difficult to gain access to places for exercise, the practice of healthy behavior was low regardless of age (group over 70 years (22.3%), group under 50 years and over 70 years (25.5%), and group under 50 years (33.1%)). Those who had easy access to places for exercise were over 50 years old, had fair SRH, had no spouse, had high blood pressure, and lived in a house all had a lower amount of healthy practices (28.3%). In addition, 30.5% of the subjects living in a place with easy access to places for exercise and a population of less than 300,000 showed a greater number of healthy practices. In contrast, 32.9% of those who had easy access to places for exercise, were over 50 years old, had fair SRH, had a spouse, had a job, and lived in a region with a population of less than 100,000 showed a higher amount of healthy practices (Figure 4).

#### 3.2.3. Importance of Factors Affecting Healthy Behavior Practices

The importance of the variables for men was in the following order: age, job, access to places for exercise, SRH, stress, city size, BMI, residential area (“*dong*” as urban areas and “*eup/myeon*” as rural areas), diabetes, and perceived environment (green space). For women, the order was: access to places for exercise, age, job, SRH, living alone, city size, housing type, education level, and hypertension (Table 2).

## 4. Discussion

Men and women had below-average healthy behavior practices if they had poor access to places for exercise and fair SRH. The healthy practices of men were lower in the group without a job, in their 40s–50s, living in a rural area, and with a lot of stress. The healthy practices of women living in less populated cities (<100 thousand of population) were also low.

Several factors influence participation in physical activity (PA), and studies have recently focused on the role of the environment in promoting healthy behaviors as determined by PA [22,23,24,25]. A short distance to urban green spaces and the availability of exercise equipment are positively associated with the frequency of PA [26]. The environment can impact healthy adults’ capacity to perform health-enhancing PA [22], while perceived green space characteristics are strong predictors of wellbeing [27]. In this study, healthy behavior was also below average in the cases where access to places for exercise was difficult. The strategies for increasing PA using place-based interventions were more common than those using person-based interventions [22,28]. Place-level interventions included changing the physical structure of parks (i.e., adding walking trails) to facilitate physical activity [29]. To increase healthy behaviors among both males and females, it is necessary to improve the access to places for exercise since the practice of physical activity is greatly influenced by the environment of the residential areas [30]. When the neighborhood exercise environment and public facilities were sufficient, there was a correlation with the SRH [31]. Kang et al. [32] found that SRH and social support had a positive correlation with healthy behavior: the more positive the SRH regardless of disease, the better it predicted individual wellbeing [33]. In this study, healthy behavior was also below-average in the cases of fair–poor SRH.

It is generally said that the search for specific ways to cope with high stress among men is insufficient [34], but in this study, high stress led to decreased health practices, so a program for stress management is needed.

Healthy behavior practices were lower in males and females when they were older. According to the results of a previous study, as age increases, the prevalence of chronic diseases also increases due to a decrease in physical activity and also to habit-based health risk behaviors [35,36,37]. In particular, rural elderly people experience more socioeconomic inequality and social exclusion than urban elderly people, and their standard of living during old age is poor [38]. Groups with low socioeconomic status are more sensitive to local resources because they lack other alternative health resources [39]. Older people are more affected by local resources than younger people because they stay in residential areas for a longer time and spend more time using local facilities or spaces [31]. The job variable was significantly associated with healthy behavior. In particular, there were more men in the job group than women, and they also had more regular walking groups. If women are significantly less likely to work outside of the home, this may explain the difference in the results between men and women. The impacts of physical and social environmental factors on behavior have been increasingly recognized. Individuals are influenced not only by their motivation and capability to make behavioral changes, but also by opportunities afforded by their social and physical environment [40,41]. Even though factors such as information and skills training are important for behavioral changes, other behavior-influencing factors should also be considered, e.g., social support [3,42].

Health inequality by region was not any individual’s fault but was caused by an unequal distribution of health protection factors and factors that are harmful to one’s health in the region [43]. If the living environment—such as the neighborhood space or the local government’s socioeconomic capabilities and policies—was health-friendly, it directly and positively affected health [44]. In other words, an individual’s health-related behavior is directly or indirectly influenced by the socioeconomic, built environment, and policy characteristics of the region, and by the operation of local health services throughout the region [45]. In previous studies, there was a difference in health behavior according to the area of residence [46,47,48,49], and in this study, both men and women were affected by access to local exercise facilities and the type of residential areas (urban, rural, etc.).

Therefore, cultivating elderly-friendly physical environments in each region and developing health programs based on supportive social activities for community residents would contribute to promoting healthy lifestyle practices. The results of this study highlight the need to implement policies and strategies that are tailored to personal and environmental factors in order to boost healthy lifestyle practices in older adults [50].

Individual health behaviors (smoking, drinking, exercise, nutrition, etc.) tend to form clusters [6,51]. Therefore, it is necessary to implement a health project with the use of a comprehensive index rather than approaching each health behavior individually.

A limitation of this study is that it was difficult to explain the temporal relationship because KCHS is a cross-sectional study. Additional limitations include the lack of many different variables using secondary data. Self-reported health behavior data (smoking, drinking, walking) may not provide accurate information because of recall and social desirability biases. These limitations may influence the interpretation of the study findings.

Despite these limitations, this study has several strengths. We used a nationally representative sample in this study, which provided evidence for the development of implementation strategies that consider not only individual characteristics, but also access to local exercise facilities and residential area characteristics (urban, rural) for the low health behavior practice group. In addition, this was the first study to use a decision tree analysis, suggesting that health projects that enhance health behaviors should be carried out, giving priority to those who are old, have no job, and have poor perceived health levels. In particular, a project aimed at improving access to local exercise facilities should be implemented.

## 5. Conclusions

It is necessary to implement an intervention on health behavior practices that considers not only individual characteristics (age, job, self-rated health status), but also access to local exercise facilities and residential area characteristics (urban, rural). In particular, the elderly and those who have a job with poor health status should be prioritized as the target groups of the health project, and for women, the group with low access to community exercise facilities should also be considered as a priority target group for the health project.

## Figures and Tables

**Table 1:** General characteristics of the subject.

Characteristics			Total (n = 16,519)	Men (n = 7217)	Women (n = 9302)	*p*-Value
Demographic	Age	19–29	1182 (7.2)	613 (8.5)	569 (6.1)	<0.001
		30–39	1758 (10.6)	801 (11.1)	957 (10.3)	
		40–49	2537 (15.4)	1209 (16.8)	1328 (14.3)	
		50–59	3157 (19.1)	1450 (20.1)	1707 (18.4)	
		60–69	3423 (20.7)	1503 (20.8)	1920 (20.6)	
		≥70	4462 (27.0)	1641 (22.7)	2821 (30.3)	
	Income ^1^	<KRW 2.0 million	6848 (41.5)	2564 (35.5)	4284 (46.1)	<0.001
		≥KRW 2.0 million	9671 (58.5)	4653 (64.5)	5018 (53.9)	
	Job	Unemployed	6544 (39.6)	1921 (26.6)	4623 (49.7)	<0.001
		Employed	9975 (60.4)	5296 (73.4)	4679 (50.3)	
	Education	≤Middle school	5255 (31.8)	1403 (19.4)	3852 (41.4)	<0.001
		≥High school	11,264 (68.2)	5814 (80.6)	5450 (58.6)	
	Marital status	Living alone	5344 (32.4)	1740 (24.1)	3604 (38.7)	<0.001
		married	11,175 (67.6)	5477 (75.9)	5698 (61.3)	
Health-related	Self-rated health status	Fair or poor	11,447 (69.3)	4597 (63.7)	6850 (73.6)	<0.001
		Good	5072 (30.7)	2620 (36.3)	2452 (26.4)	
	Stress	Low	13,148 (79.6)	5881 (81.5)	7267 (78.1)	<0.001
		High	3371 (20.4)	1336 (18.5)	2035 (21.9)	
	Perceived depression	No	15,696 (95.0)	6981 (96.7)	8715 (93.7)	<0.001
		Yes	823 (5.0)	236 (3.3)	587 (6.3)	
	Body Mass Index (m^2^/kg)	Underweight	642 (3.9)	239 (3.3)	403 (4.3)	<0.001
		Normal	10,117 (61.2)	4196 (58.1)	5921 (63.7)	
		Obesity	5760 (34.9)	2782 (38.5)	2978 (32.0)	
	Hypertension	No	11,673 (70.7)	5343 (74.0)	6330 (68.0)	<0.001
		Yes	4846 (29.3)	1874 (26.0)	2972 (32.0)	
	Diabetes mellitus	No	14,665 (88.8)	6396 (88.6)	8269 (88.9)	0.584
		Yes	1854 (11.2)	821 (11.4)	1033 (11.1)	
Region	Housing type	House	11,079 (67.1)	4764 (66.0)	6315 (67.9)	0.011
		Apartment	5440 (32.9)	2453 (34.0)	2987 (32.1)	
	Residential area ^2^	Urban	5757 (34.9)	2564 (35.5)	3193 (34.3)	0.108
		Rural	10,762 (65.1)	4653 (64.5)	6109 (65.7)	
	City size ^3^	City 1	5062 (30.6)	2259 (31.3)	2803 (30.1)	0.053
		City 2	3386 (20.5)	1514 (21.0)	1872 (20.1)	
		City 3	3280 (19.9)	1422 (19.7)	1858 (20.0)	
		City 4	4791 (29.0)	2022 (28.0)	2769 (29.8)	
	Access to places for exercise	Difficulty	4149 (25.1)	1741 (24.1)	2408 (25.9)	0.010
		Easy	12,370 (74.9)	5476 (75.9)	6894 (74.1)	
	Perceived environment (atmosphere)	Bad	5520 (33.4)	2397 (33.2)	3123 (33.6)	0.626
		Good	10,999 (66.6)	4820 (66.8)	6179 (66.4)	
	Perceived environment (green space)	Bad	4886 (29.6)	2119 (29.4)	2767 (29.7)	0.591
		Good	11,633 (70.4)	5098 (70.6)	6535 (70.3)	
Healthy behavior ^4^		No	11,703 (70.8)	5629 (78.0)	6074 (65.3)	<0.001
		Yes	4816 (29.2)	1588 (22.0)	3228 (34.7)	
Smoking status		No	13,824 (83.7)	4761 (66.0)	9063 (97.4)	<0.001
		Yes	2695 (16.3)	2456 (34.0)	239 (2.6)	
High-risk drinking ^5^		No	14,850 (89.9)	5548 (76.9)	9302 (100)	<0.001
		Yes	1669 (10.1)	1669 (23.1)	0 (0.0)	
Regular walking ^6^		No	10,519 (63.7)	4513 (62.5)	6006 (64.6)	0.007
		Yes	5999 (36.3)	2704 (37.5)	3295 (35.4)	
Total			16,519 (100.0)	7217 (43.7)	9302 (56.3)	

Values are presented as numbers (%). *p*-values were determined by chi-square test. ^1^ Unit of currency: Korean won (KRW); KRW 1.0 million is approximately USD 1000. ^2^ “Dong” as urban area and “eup/myeon” as rural area. ^3^ City 1: ≥300 thousand population, city 2: <300 thousand and ≥100 thousand population, city 3: <100 thousand and ≥50 thousand population, city 4: <50 thousand population. ^4^ Healthy behavior was defined as engagement in all three healthy behaviors (non-smoking, not-high-risk drinking, and regular walking). ^5^ High-risk drinking; individuals who had more than seven (for men) or five (for women) drinks on the same occasion on at least 2 of the past 7 days. ^6^ Regular walking; participating in walking activities for at least 30 min, five or more days a week.

**Table 2:** Evaluation of the importance of independent variables.

	Variables	Importance
Men	Age	0.46
	Job	0.15
	Access to places for exercise	0.13
	Self-rated health status	0.11
	Stress	0.07
	City size ^1^	0.02
	Body Mass Index	0.02
	Residential area	0.02
	Diabetes mellitus	0.02
	Perceived environment (green space)	0.01
Women	Access to places to exercise	0.33
	Age	0.28
	Job	0.08
	Self-rated health status	0.07
	Living alone	0.07
	City size ^2^	0.07
	Housing type	0.04
	Education	0.03
	Hypertension	0.03

^1^ City size: City 1 (≥300,000 population). ^2^ City size: City 2 (<300,000 and ≥100,000 population).

## Data Availability

Publicly available datasets were analyzed in this study. These data can be found here: https://chs.kdca.go.kr/chs/rawDta/rawDtaProvdMain.do; we accessed the data on 24 December 2019.
